# Supplementary material for: Inhibitory Effects of Roseoside and Icariside E4 Isolated from a Natural Product Mixture (No-ap) on the Expression of Angiotensin II Receptor 1 and Oxidative Stress in Angiotensin II-Stimulated H9C2 Cells
Source: Molecules. 2019 Jan 23;24(3):414. doi: 10.3390/molecules24030414 (PMC6384670; doi:10.3390/molecules24030414)
Supplement: Supplementary file 1 [file molecules-24-00414-s001.pdf]

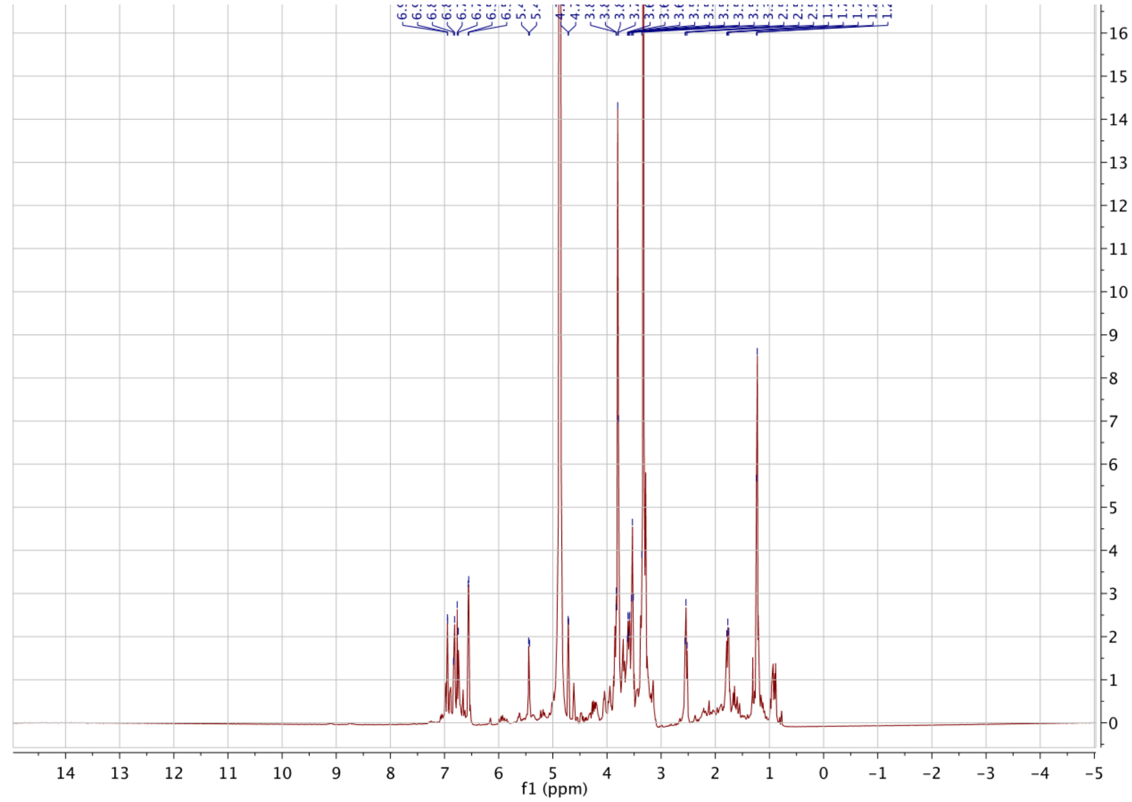

Figure S1. ^{1}H-NMR spectra of Roseoside (1)

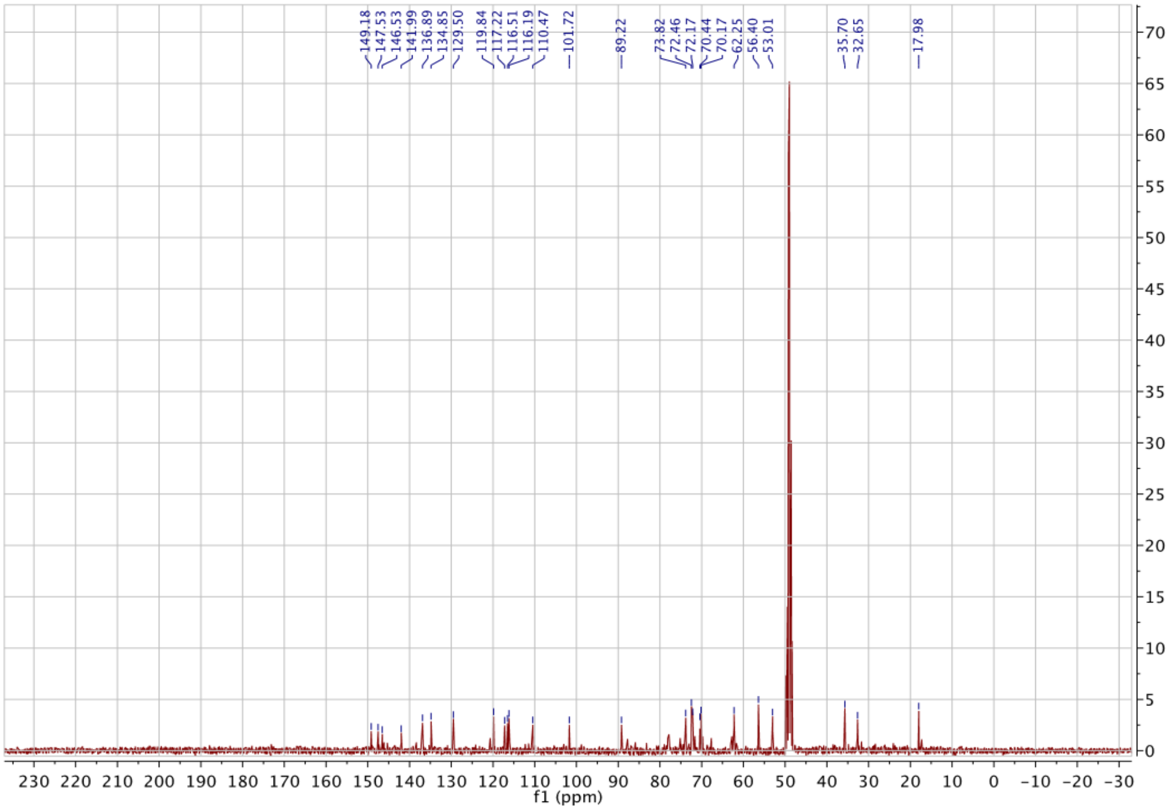

Figure S2. ^{13}C-NMR spectra of Roseoside (1)

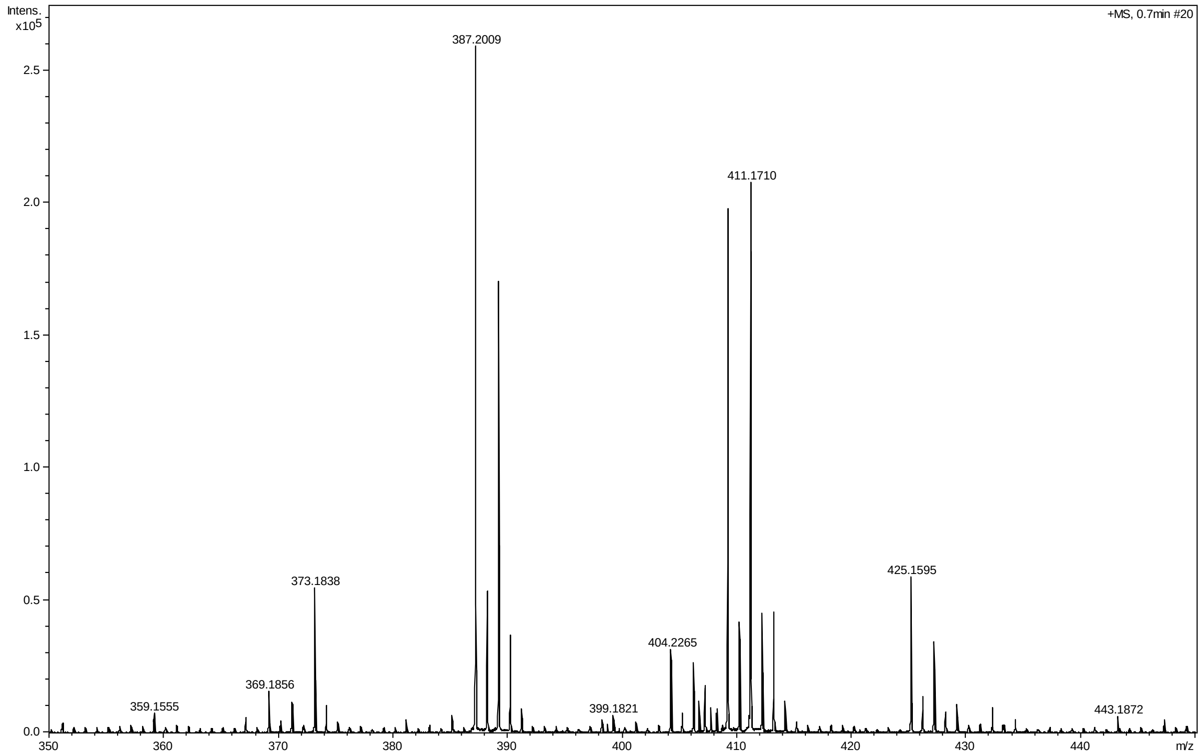

Figure S3. ESI-MS spectra of Roseoside (1)

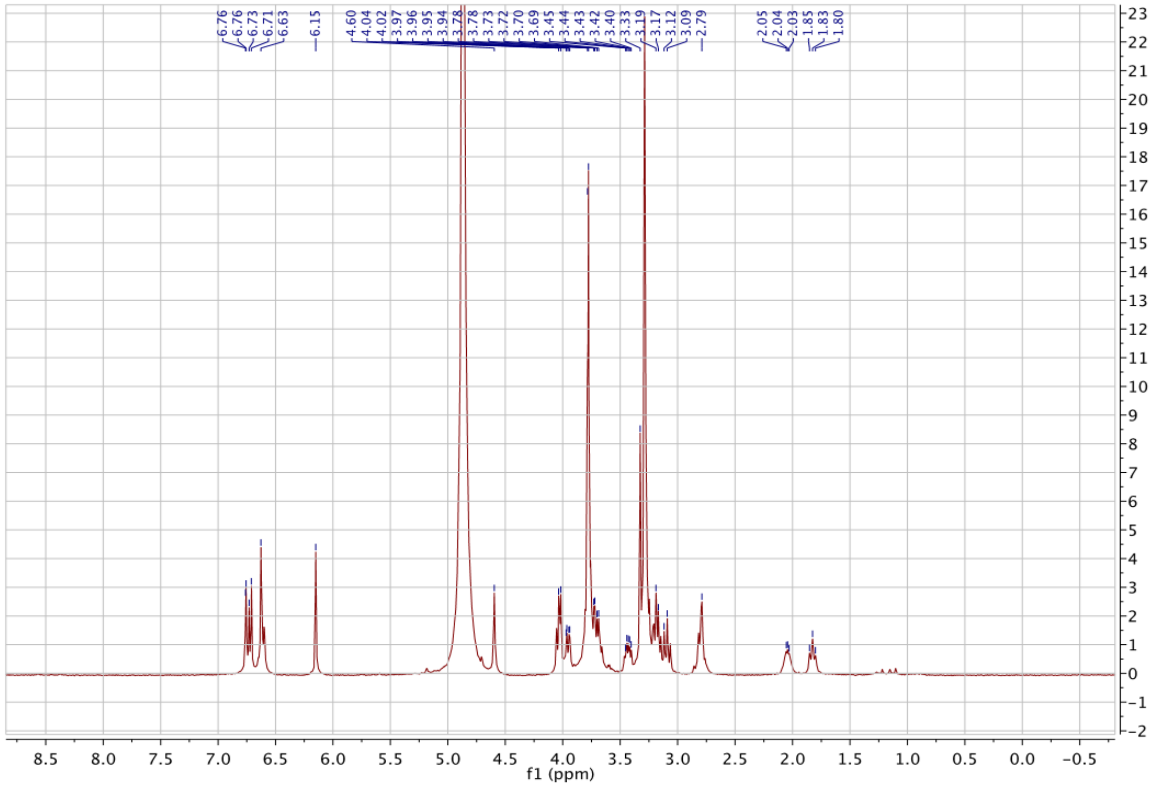

Figure S4. ^{1}H-NMR spectra of Isolariciresinol 9-O-β-D-xyloside (2)

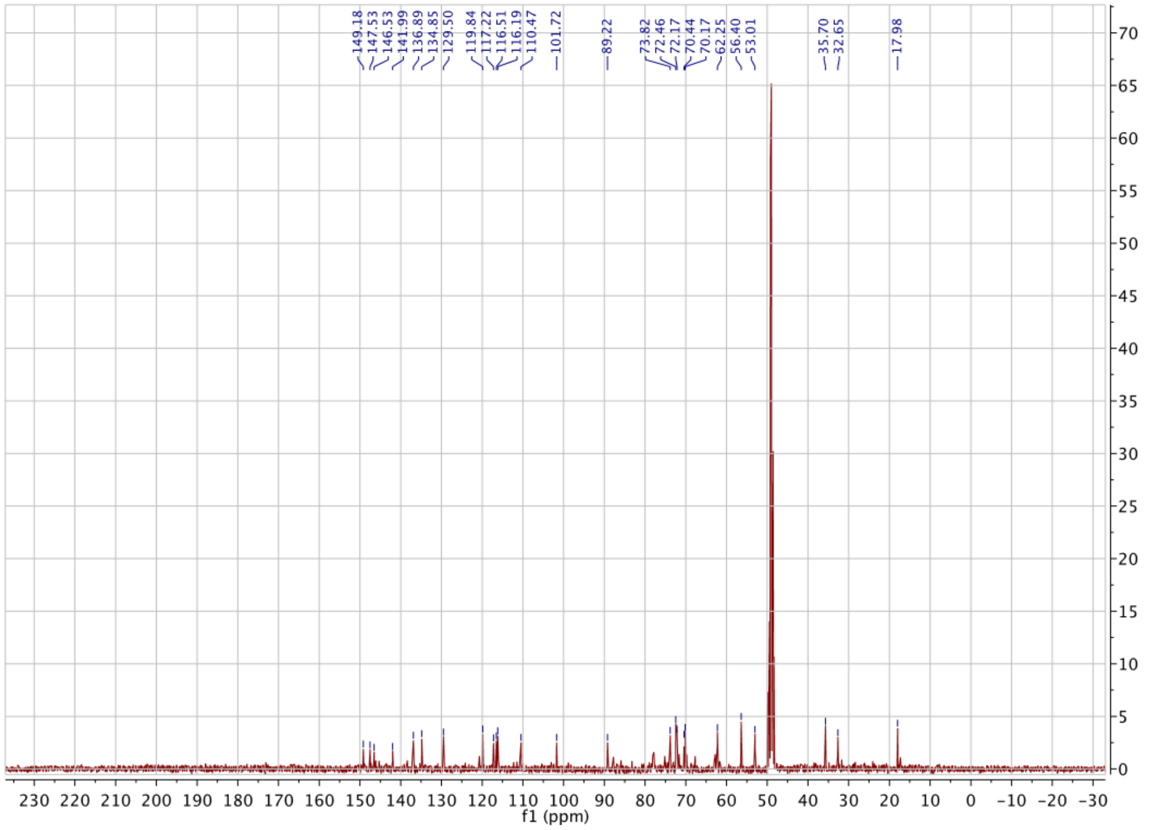

Figure S5. ^{13}C-NMR spectra of Isolariciresinol 9-O-β-D-xyloside (2)

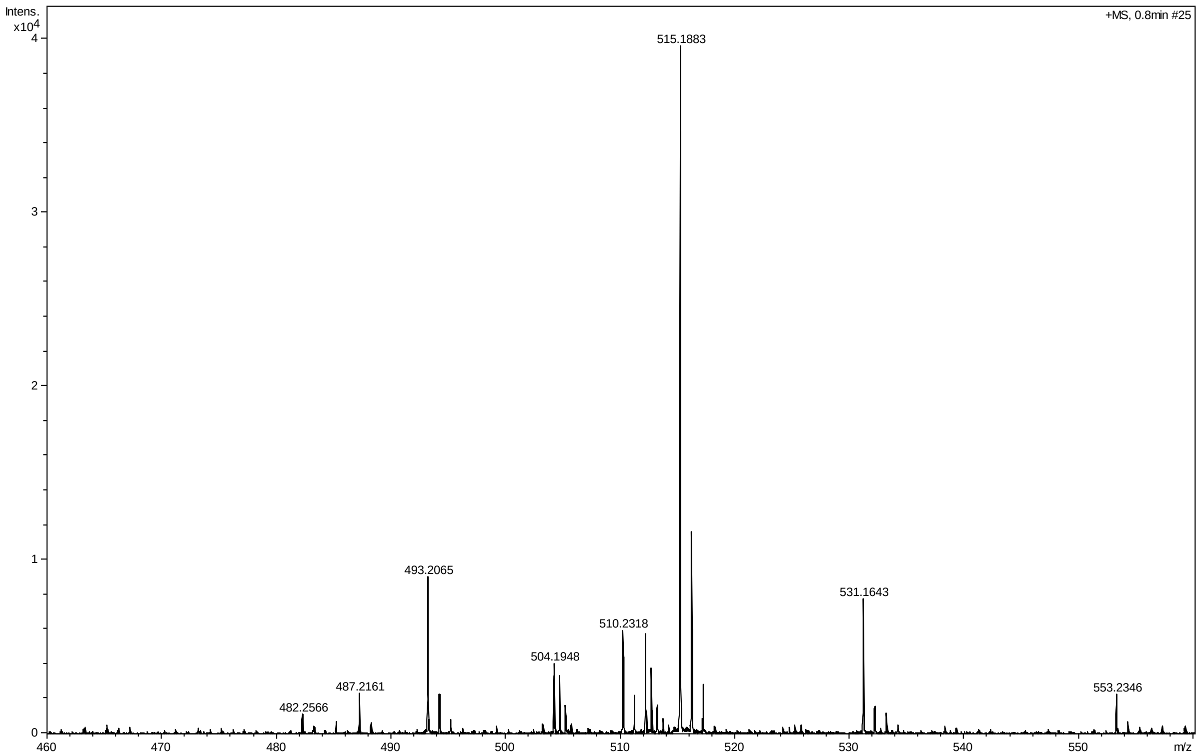

Figure S6. ESI-MS spectra of Isolariciresinol 9-O-β-D-xyloside (2)

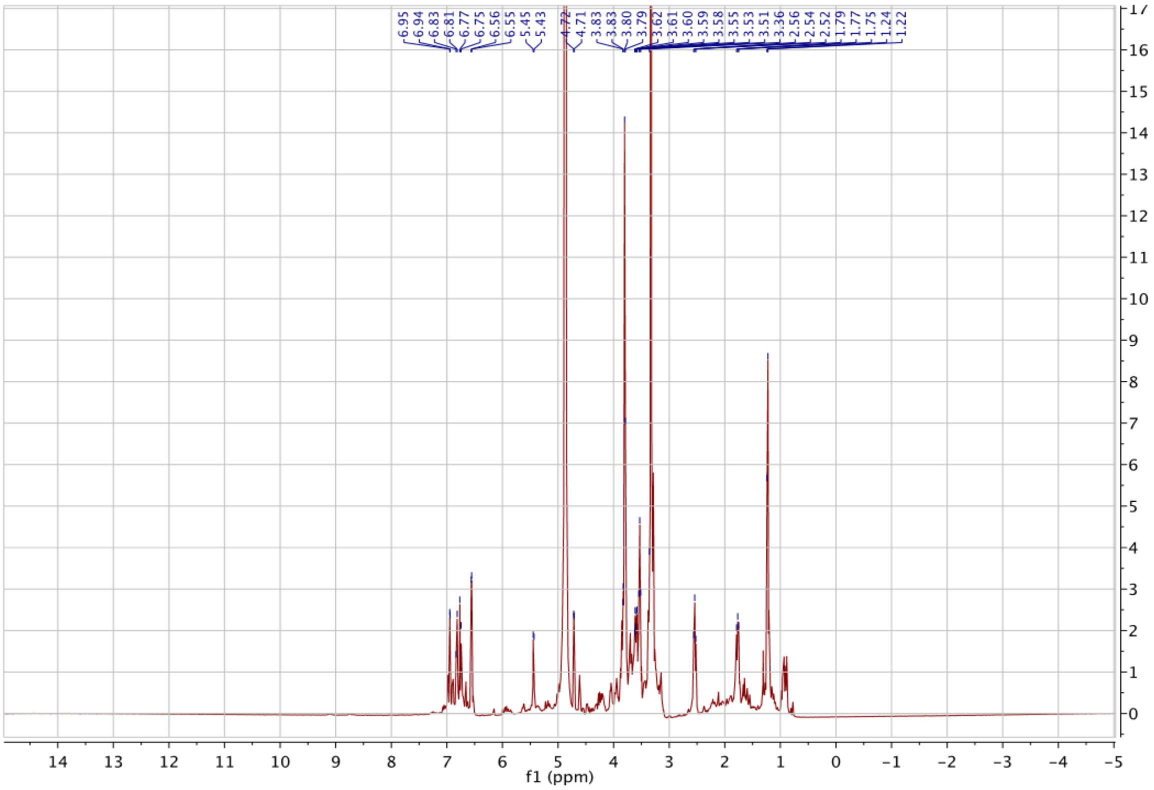

Figure S7. ^{1}H-NMR spectra of Massonianoside B (3)

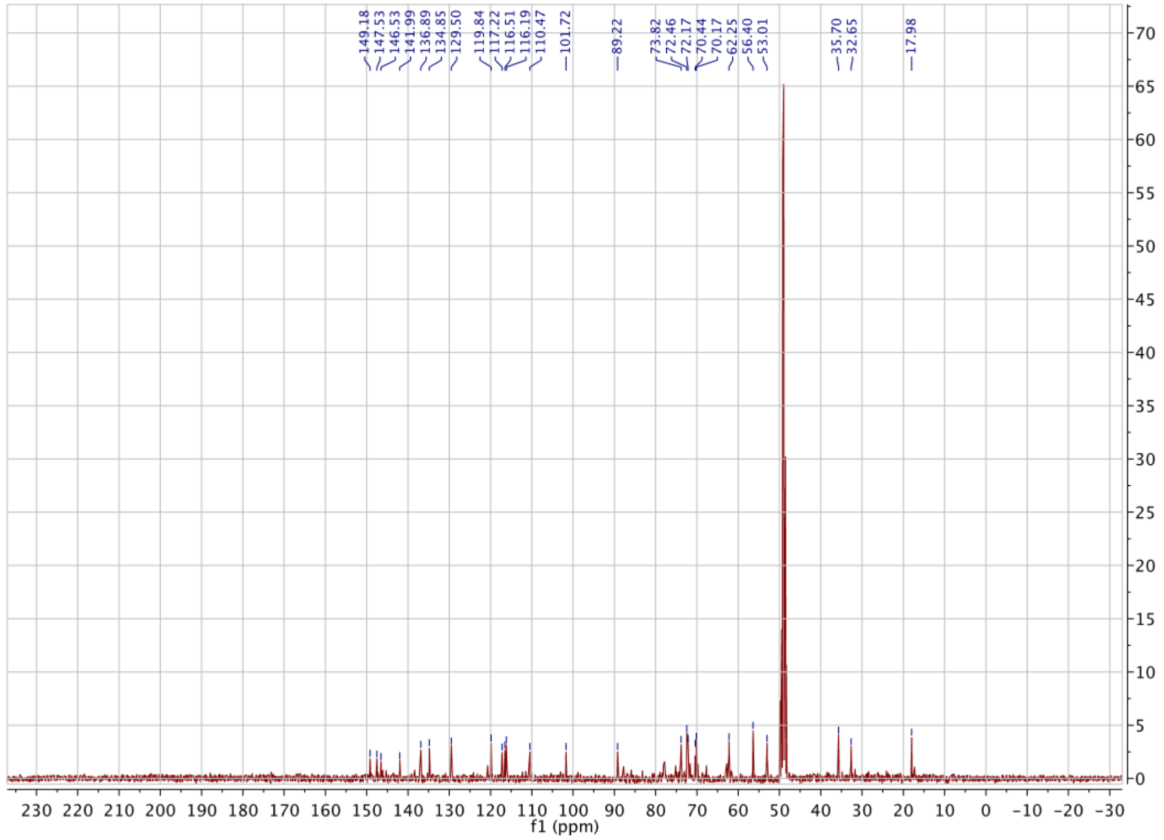

Figure S8. ^{13}C-NMR spectra of Massonianoside B (3)

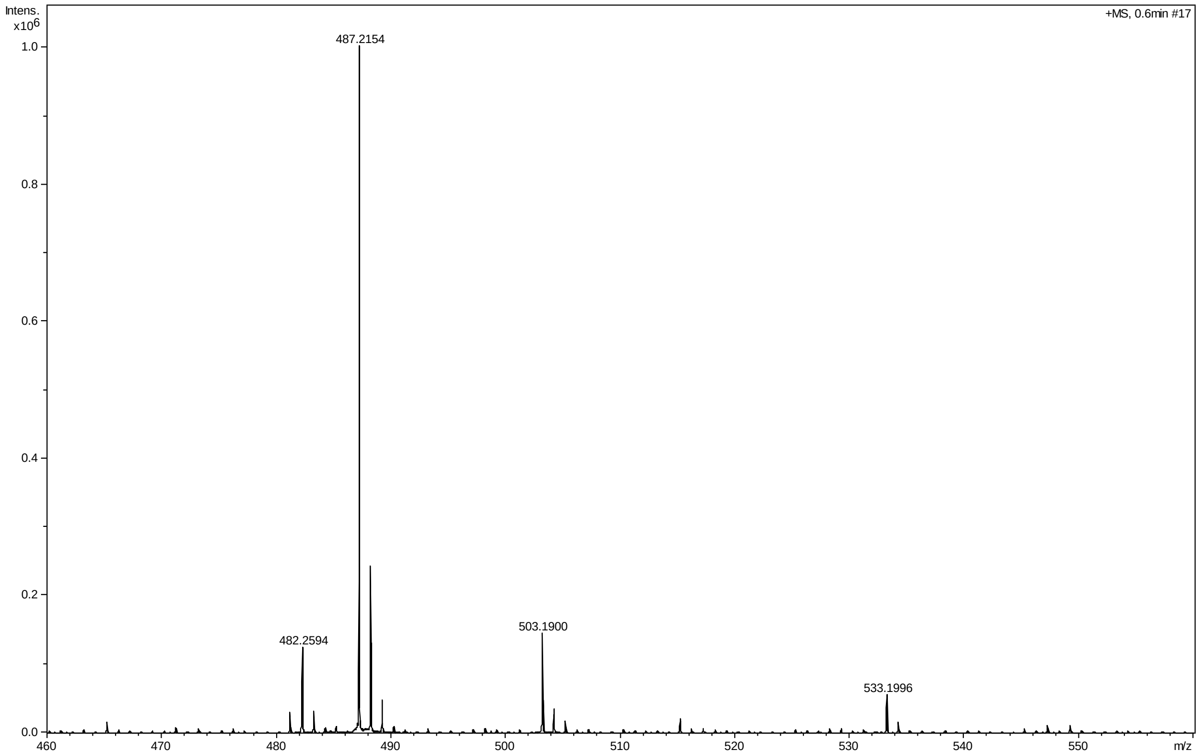

Figure S9. ESI-MS spectra of Massonianoside B (3)

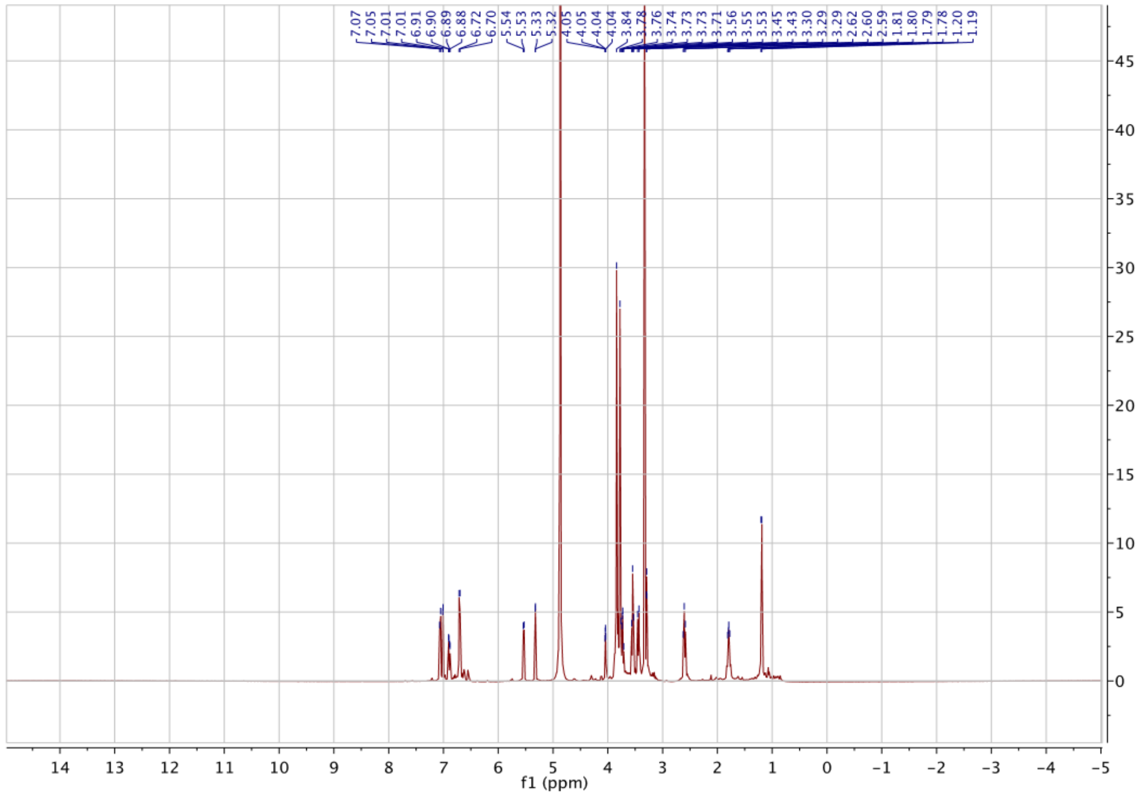

Figure S10. ^{1}H-NMR spectra of Icariside E4 (4)

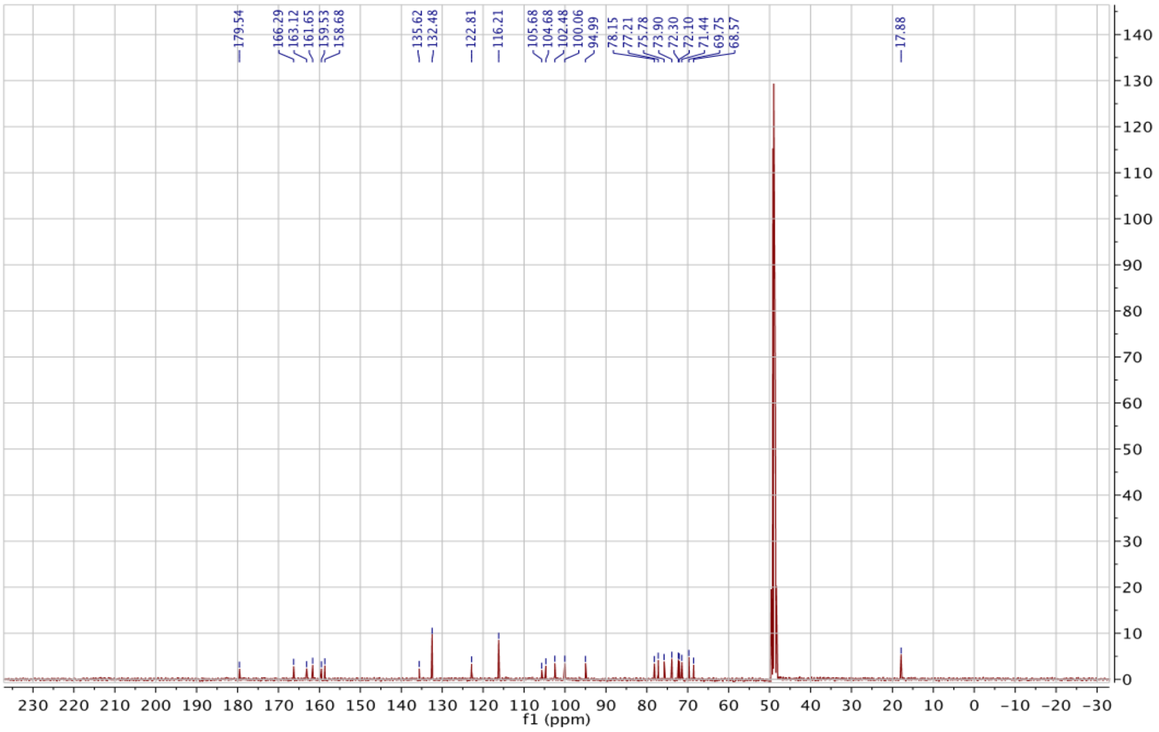

Figure S11. ^{13}C-NMR spectra of Icariside E4 (4)

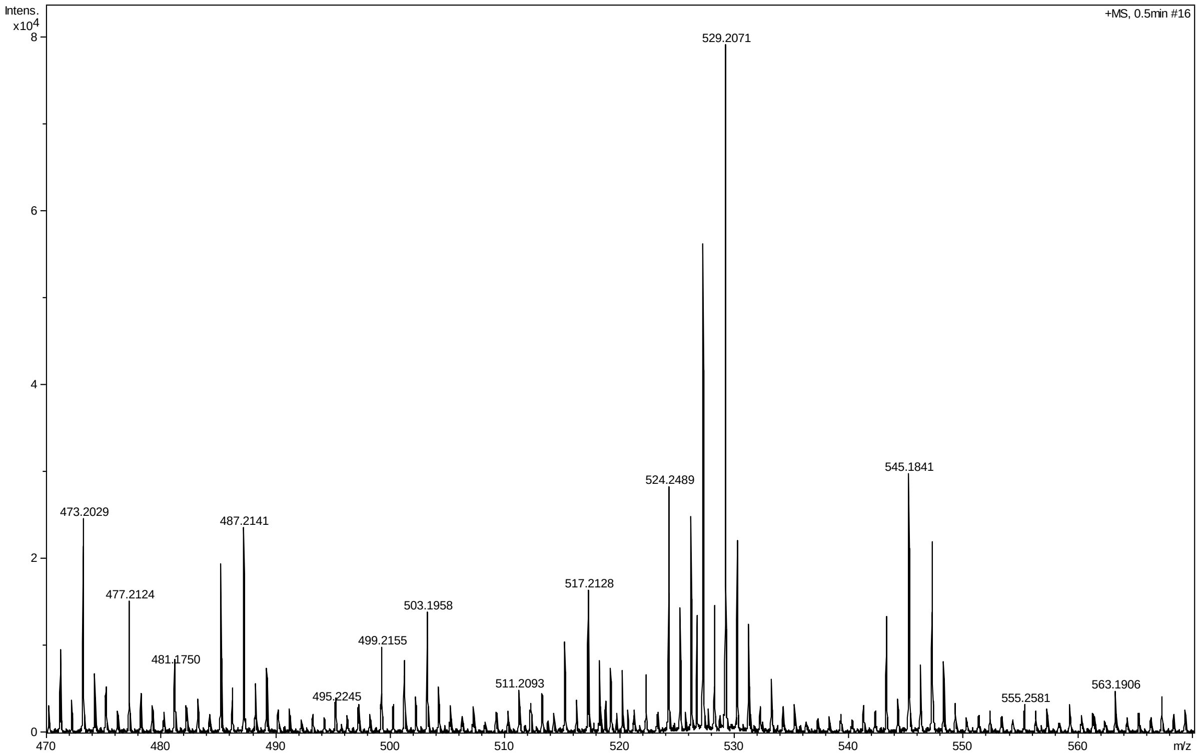

Figure S12. ESI-MS spectra of Icariside E4 (4)

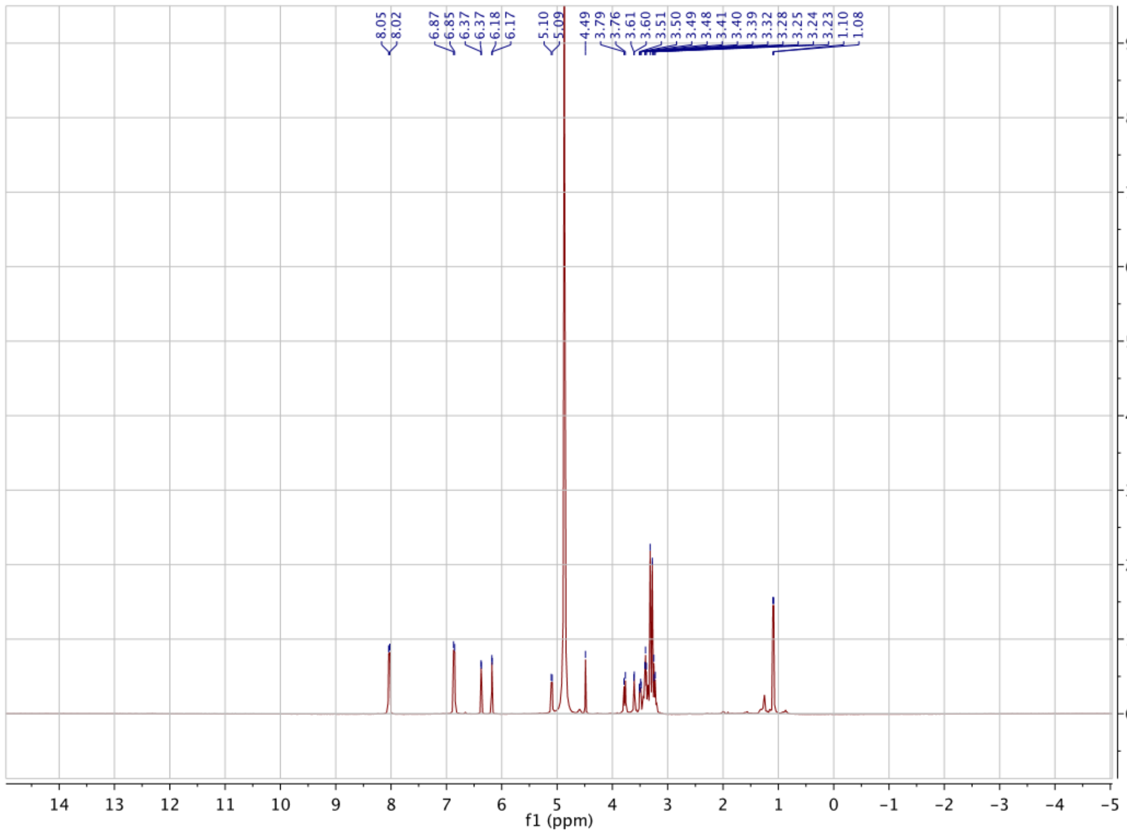

Figure S13. ^{1}H-NMR spectra of Nicotiflorin (5)

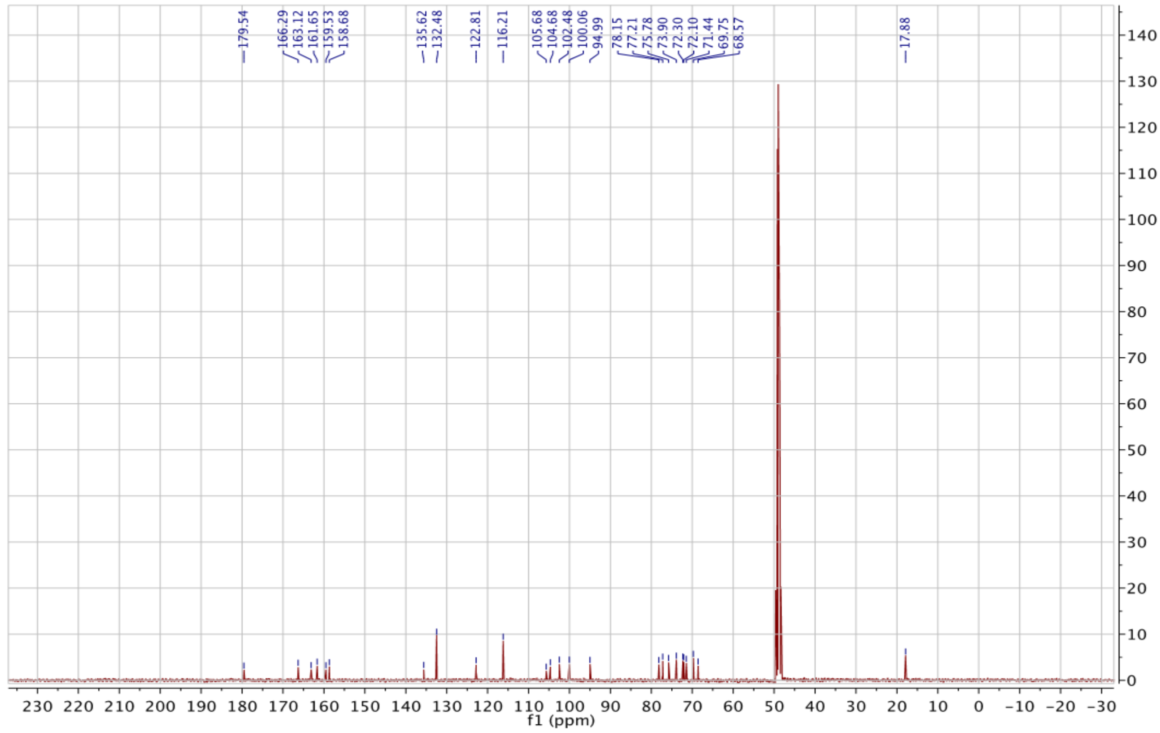

Figure S14. ^{13}C-NMR spectra of Nicotiflorin (5)

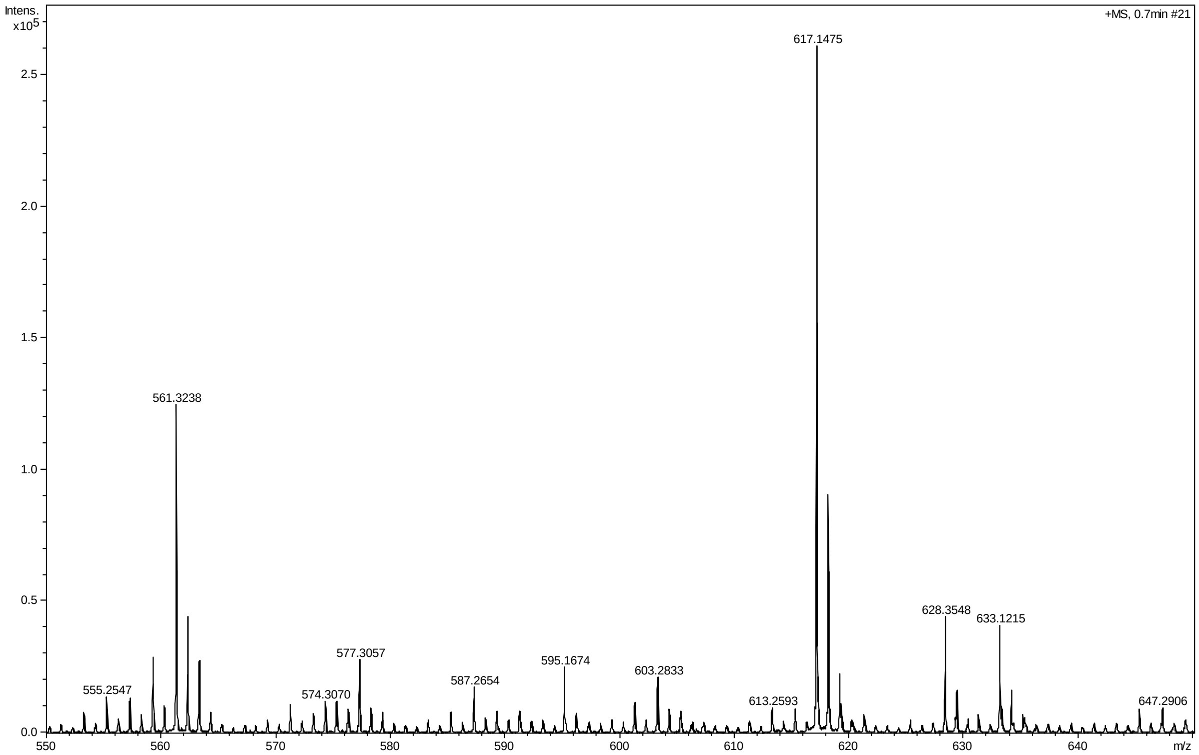

Figure S15. ESI-MS spectra of Nicotiflorin (5)
